# A Flexible Artificial Sensory Nerve Enabled by Nanoparticle‐Assembled Synaptic Devices for Neuromorphic Tactile Recognition

**DOI:** 10.1002/advs.202106124

**Published:** 2022-06-09

**Authors:** Chengpeng Jiang, Jiaqi Liu, Lu Yang, Jiangdong Gong, Huanhuan Wei, Wentao Xu

**Affiliations:** ^1^ Institute of Photoelectronic Thin Film Devices and Technology College of Electronic Information and Optical Engineering Nankai University Tianjin 300350 P. R. China; ^2^ Research Center for Intelligent Sensing Zhejiang Lab Hangzhou 311100 P. R. China

**Keywords:** flexible synaptic electronics, nanomaterial self‐assembly, neuromorphic perception, robotics and smart interfaces, tactile recognition

## Abstract

Tactile perception enabled by somatosensory system in human is essential for dexterous tool usage, communication, and interaction. Imparting tactile recognition functions to advanced robots and interactive systems can potentially improve their cognition and intelligence. Here, a flexible artificial sensory nerve that mimics the tactile sensing, neural coding, and synaptic processing functions in human sensory nerve is developed to achieve neuromorphic tactile recognition at device level without relying on algorithms or computing resources. An interfacial self‐assembly technique, which produces uniform and defect‐less thin film of semiconductor nanoparticles on arbitrary substrates, is employed to prepare the flexible synaptic device. The neural facilitation and sensory memory characteristics of the proton‐gating synaptic device enable this system to identify material hardness during robotic grasping and recognize tapping patterns during tactile interaction in a continuous, real‐time, high‐accuracy manner, demonstrating neuromorphic intelligence and recognition capabilities. This artificial sensory nerve produced in wearable and portable form can be readily integrated with advanced robots and smart human–machine interfaces for implementing human‐like tactile cognition in neuromorphic electronics toward robotic and wearable applications.

## Graphical abstract

A flexible artificial sensory nerve that achieves neuromorphic tactile recognition in a real‐time and near‐sensor manner is developed. In this system, tactile signals coded as spike trains are processed by synaptic devices with sensory memory, and tactile recognitions are performed at device level by directly using device output without relying on computing resources or algorithms.

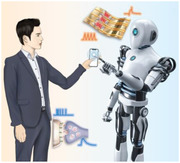

## 1 Introduction

Tactile perception and tactile recognition in human involve assessments of strength, distribution, and patterns of sensory stimulations and events imposed on the skin by active or passive touch.^[^ ^1^, ^2^ ^]^ The process is that external stimuli received by sensory receptors in the skin are encoded as neural spikes and processed by neurons and synapses using intelligent functions including adaptation, filtering, amplification, and memory, and then transmitted to the cerebral cortex for achieving high‐level functions, such as classification, identification, and perceptual learning.^[^ ^3^, ^4^, ^5^ ^]^ Such sophisticated tactile perception allows humans to perform complex tasks such as delicate grasping, texture discrimination, and object identification by perceiving tactile features and tactile patterns through touching and interacting.^[^ ^2^, ^6^ ^]^ Imparting tactile recognition functions to robots, prosthetics, and interactive systems could potentially improve their cognition and intelligence when interacting with unstructured environments and manipulating unknown objects.^[^ ^7^, ^8^, ^9^, ^10^ ^]^

Recent advances in neuromorphic electronics have boosted the development of artificial tactile sensory systems.^[^ ^11^, ^12^ ^]^ For instance, an artificial afferent nerve composed of pressure sensor arrays and organic synaptic transistors can distinguish Braille characters by frequency analysis, and an optoelectronic afferent nerve implemented by encoding sensor signals as optical spikes can detect touch and recognize handwriting with the help of software algorithm.^[^ ^3^, ^13^ ^]^ By processing tactile data using synaptic device and machine learning algorithm, recognition of contacting and writing patterns have been realized in an artificial sensory nerve that is composed of synaptic transistors and memristor arrays.^[^ ^14^, ^15^ ^]^ An artificial tactile skin device built from pressure/vibration responsive tactile sensor can predict and classify fabric textures by using deep learning, in which adaptive neural signal processing was implemented in a neural circuit board with multiple processors.^[^ ^16^ ^]^ These reported sensory systems achieve sensory processing of tactile stimuli in the form of neural spikes. However, their synaptic electronics requires additional computing or processing units, and tactile recognition tasks are not performed in a real‐time and near‐sensor manner, given that the recognition functions are implemented either in offline simulation or with the aid of machine learning algorithms. Future development of wearable sensory systems and wireless sensory networks requires near‐sensor or even in‐sensor intelligence to allow real‐time sensory processing with low latency and low computational cost. In this sense, there is a great need to develop artificial tactile sensory systems with intrinsic capabilities of neuromorphic perception and recognition, preferably implemented at the device level.^[^ ^12^, ^17^ ^]^ Such a system would benefit the realization of human‐like tactile cognition in neuromorphic electronics, and may find applications in e‐skin, smart interfaces, and advanced robotics to help achieve tactile tasks such as dexterous manipulation, object identification, and tactile interaction.^[^ ^8^, ^10^, ^18^ ^]^

In this work, we reported a flexible artificial sensory nerve that achieves neuromorphic tactile recognition in a real‐time and near‐sensor manner at the device level. A flexible tactile sensor showing linear response and high sensitivity was used for tactile sensing, while a flexible synaptic transistor exhibiting neural facilitation and sensory memory was used for sensory processing. Notably, the synaptic device was fabricated using a facile and scalable technique of nanoparticle (NP) self‐assembly, which can produce a uniform and continuous film of assembled NPs at arbitrary substrates. Tactile recognition was performed by directly reading the device output without relying on algorithms or computing resources, and the synaptic device requires no operation of state resetting or memory erasing. Such implementation simplifies the architecture of artificial sensory system while enhancing its neuromorphic perception capabilities. Identification of material hardness during robotic grasping and recognition of tapping patterns during tactile interaction were both demonstrated to prove the tactile recognition capabilities of our system. This work provides a guideline for developing neuromorphic sensory system with human‐like cognition toward emerging applications, including brain‐inspired artificial intelligence (AI), sensory robotics, and interactive electronic skin, which require real‐time perception and efficient recognition.

## 2 Results and Discussion

### 2.1 Flexible Artificial Sensory Nerve

**Figure 1:**
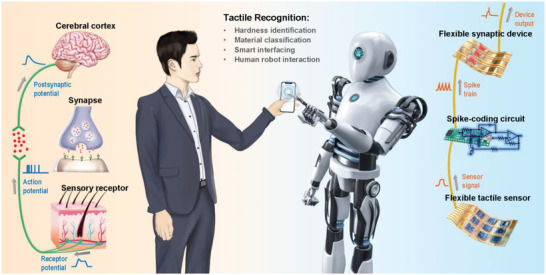
Schematic illustration of the flexible artificial sensory nerve (right part) that mimics the biological tactile sensory system (left part) toward neuromorphic tactile recognition. In this system, tactile signals coded as spike trains are processed by synaptic devices with sensory memory, and tactile recognitions are achieved in a real‐time and near‐sensor manner by directly using device output without relying on computing resources or algorithms.

Our flexible artificial sensory nerve process information in a way that resembles the signal‐transmission and signal‐processing characteristics in a biological tactile sensory system by using simple circuits and synaptic electronics to implement neural coding and neural processing (**Figure** **1**). Sensory receptors such as mechanosensitive Merkel cells distributed in the skin, which perceive tactile stimuli and produce receptor potential, are emulated by a resistive‐type tactile sensor. Pressure applied by touch causes resistance change in the flexible tactile sensor, leading to the generation of a continuous analog signal. This time‐dependent sensor signal is further converted to rate‐coded spike trains by the spike‐encoding circuit, in a way similar to the sensory neuron in an afferent nerve that generates action potentials.^[^ ^5^ ^]^ When the flexible synaptic transistor receives the spike trains, its channel conductivity is modulated, and its drain current is output as postsynaptic current (PSC). This process mimics the signal‐processing function of a synapse connected to neurons by dendritic integration, in which action potential causes release of neurotransmitters and further stimulates the postsynaptic potentials.^[^ ^5^ ^]^ A biological tactile sensory system also involves cerebral cortex in the brain for analyzing and recognizing multimodal tactile information by touch, perception, and learning.^[^ ^6^ ^]^ In our system, device outputs including spike pulse number and synaptic weight are used as classification criteria for real‐time intelligent tactile recognition in robotic manipulation and tactile interaction. Furthermore, the tactile sensor and the synaptic device in our system are both flexible and can be integrated with other wearable electronics or robotic systems.^[^ ^19^ ^]^

### 2.2 Flexible Synaptic Device

**Figure 2:**
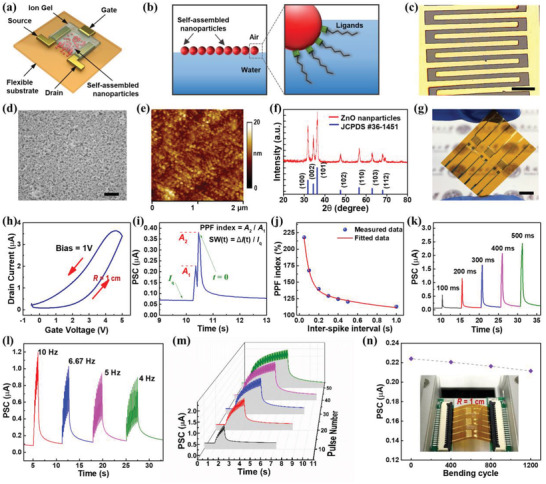
Performance of the flexible synaptic device. a) Schematic illustration of device structure. b) Schematic illustration of NP self‐assembly at air/water interface. c) Optical image, d) SEM image, e) AFM image, and f) XRD pattern of the self‐assembled NP film fabricated as transistor channel on Si/SiO_2_ substrate. g) Photograph of flexible synaptic transistor array fabricated on polyimide substrate. h) Transfer curve of the device under *V*_ds_ = 1 V. i) PSC of the device triggered by two pulses (5 V, 50 ms). Synaptic weight (SW) and paired pulse facilitation (PPF) ratio are explained schematically. j) PPF index as a function of interspike interval. k) PSC of the device as a function of spike duration, l) spike frequency, and m) spike number, showing spike duration‐dependent plasticity (SDDP), spike rate‐dependent plasticity (SRDP), and spike number‐dependent plasticity (SNDP) characteristics respectively. n) PSC of the device under repeated bending over 1200 cycles. Inset: photograph of the device under bending. The scale bar in (c), (d), and (g) are 500 µm, 200 nm, and 5 mm, respectively.

The synaptic device is an ion‐gel gated transistor composed of interdigitated electrodes, self‐assembled NP channel, and chitosan‐based electrolyte on a polyimide flexible substrate (**Figure** **2**a). Inorganic metal oxide nanoparticles were used as the semiconducting material for building the synaptic device due to their suitability for self‐assembly fabrication as well as their chemical stability for flexible electronics applications. The uniform and defect‐less structure of the NP channel as well as the high surface/volume ratio of the NP ensure a good interface between ion gel and channel. In‐plane gate structure was adopted to simplify device fabrication process and allow multiple presynaptic inputs. The device realized synaptic functions as a result of lateral gating by protons. As chitosan in the ion gel becomes protonated by acetic acid, the protons within the chitosan‐derived polysaccharide electrolyte accumulate at the chitosan/ZnO interface upon the arrival of positive presynaptic spikes. This process leads to formation of an electric double layer.^[^ ^20^ ^]^ Consequently, the drain current, which is regarded as the PSC, increases due to the accumulated electrons in the n‐type channel induced by proton–electron electrostatic coupling effect. After the presynaptic spike stops, the protons that accumulated at the electrolyte/channel interface gradually diffuse away, and the PSC in the channel decays to the initial value.^[^ ^21^ ^]^ This process is responsible for the transient response of excitatory postsynaptic current measured under bias voltage *V*_ds_ = 1 V when a single spike (5 V, 50 ms) was applied to the gate of the device (Figure S1, Supporting Information).

To fabricate the channel composed of semiconductor NPs, an improved technique of interfacial self‐assembly (Figure 2b) was used to enable scalable and low‐cost fabrication.^[^ ^22^, ^23^ ^]^ A floating film forms after colloidal NPs (amphiphilic ZnO NPs, Figure S2, Supporting Information) spread on an air–water interface. Ligands capped on NP surface expel NPs from the water subphase, and the NPs autonomously organize into a Langmuir film, under the mediation of surface tension and interparticle interaction.^[^ ^23^ ^]^ The self‐assembled NP film was transferred to a Si/SiO_2_ substrate for characterization. Optical microscope images (Figure 2c; Figure S3, Supporting Information) and scanning electron microscope (SEM) image (Figure 2d) reveal that this film composed of closely packed NPs is continuous and homogeneous over a large area. Atomic force microscopy (AFM) analysis of this film (Figure 2e) gives roughness of 3 nm and thickness of 25 nm, which indicate that about two layers of NPs (size 10–15 nm) were deposited. X‐ray diffraction (XRD) patterns (Figure 2f) reveal that the positions and relative intensities of the diffraction peaks are well indexed to the standard diffraction data of ZnO. The self‐assembled NP film is structurally continuous and mechanically robust, so it can be transferred directly to arbitrary surfaces. The synaptic transistor was fabricated in an array on a flexible polyimide, and the resulting device was mechanically flexible (Figure 2g).

The transfer curve of the synaptic device (Figure 2h) shows large anticlockwise hysteresis, indicating that mobile ions can effectively control the carrier density in the channel. Paired‐pulse facilitation (PPF) as a form of short‐term and activity‐dependent synaptic plasticity is common to most chemically transmitting synapses, and it is manifested by amplitude enhancement of the second of two rapidly evoked excitatory postsynaptic potentials.^[^ ^24^ ^]^ Typical PPF behavior of the device (Figure 2i) was evaluated by measuring the PSC in response to two successive presynaptic spikes (5 V, 50 ms) with interspike interval *t*_ISI_ = 100 ms. The observed facilitating effect is due to the additional proton accumulation induced by the second spike while the protons induced by the first spike have not completely diffused away.^[^ ^25^ ^]^ The PPF index describing this process is defined as: PPF index = 100% × *A*_2_/*A*_1_, where *A*_1_ and *A*_2_ are the peak PSC triggered by the first and the second spikes, respectively. Synaptic weight (SW) describing the connection strength between presynaptic and postsynaptic neurons is defined here as: SW(*t*) = Δ*I*(*t*)/*I*_q_, where Δ*I*(*t*) is the PSC current change after stimulation, *I*_q_ is the quiescent current before stimulation, and *t* = 0 corresponds to the time when presynaptic stimuli stop. PPF index increased as the interspike interval decreased (Figure 2j), and the maximum PPF index of 218% was obtained at interval of 50 ms. This trend can be well fitted by a double‐exponential function that describes two‐phase behavior in a biological synapse

(1) $$\text{PPF}\text{index}=1+C_{1}\cdot \exp\left(-t_{\mathrm{ISI}}/\tau_{1}\right)+C_{2}\cdot \exp\left(-t_{\mathrm{ISI}}/\tau_{2}\right)$$

where *C*_1_ and *C*_2_ are the facilitation magnitudes of a rapid‐decay phase (lasting tens of milliseconds) and a slow‐decay phase (lasting hundreds of milliseconds), and *τ*_1_ and *τ*_2_ are the characteristic relaxation times of these two phases.^[^ ^26^ ^]^ The fitted relaxation times (*τ*_1_ = 56 ms, *τ*_2_ = 781 ms) are comparable to those of a biological synapse.^[^ ^26^ ^]^

The device shows spike‐duration‐dependent plasticity (SDDP) (Figure 2k). PSC increases almost linearly as the duration of spike is increased (from 100 to 500 ms), and this property can be utilized for temporal coding by tuning the duty cycle of spike trains.^[^ ^27^ ^]^ The device also shows spike‐rate‐dependent plasticity (SRDP) (Figure 2l). This is another important synaptic property, in which the synaptic weight between connected neurons changes in response to the firing rate of action potentials.^[^ ^28^, ^29^ ^]^ Spike trains (5 V, 50 ms, 10 spikes) with higher frequency lead to larger synaptic weight (up to 13.9) and higher PSC (up to 1.2 µA), indicating that the device acts as a dynamic high‐pass filter. This device property is similar to the temporal filtering in synaptic transmission for neural information processing,^[^ ^1^, ^28^ ^]^ and it serves as the guideline for implementing the spike‐rate coding strategy. The device also shows spike‐number‐dependent plasticity (SNDP) (Figure 2m). Spike trains (5 V, 50 ms) with increasing spike numbers (from 10 to 50) induce increase of peak PSC. After the presynaptic spike stop, the PSC decay gradually to the level of quiescent current (*I*_q_). This decay process can be fitted to the memory loss curve

(2) $$I\left(t\right)=I_{q}+A\cdot \exp\left(-t/\tau \right)$$

where *I*(*t*), *A*, and *τ* denote the PSC, prefactor and time constant of decay, respectively.^[^ ^30^ ^]^ As spike number is increased from 10 to 50, the synaptic weight increases from to 9.7 to 20.7 and *τ* increases from 0.15 to 0.36 s. The PSC settles almost to the quiescent value within several seconds (≈5 s). The timescale of this decay process after spike‐train stimulation is similar to that of the human sensory memory (0.2–3 s), which is the perception of sensory information entering through sensory cortices and relaying through the thalamus.^[^ ^31^ ^]^ These characteristics of SDDP, SRDP, and SNDP imply that synaptic weight of the device can be regulated by pulse duration, pulse frequency, and pulse number of spike trains. Bending test was also performed by repeatedly bending the device to a radius of ≈1 cm and recording the PSC in response to a single pulse (5 V, 50 ms). The PSC shows excellent bending stability (<5% degradation) (Figure 2n), which can be attributed to the mechanical resilience of the self‐assembled NP film and the flexibility of the ion gel. This device is suitable for use in wearable neuromorphic electronics.

### 2.3 Flexible Tactile Sensor

**Figure 3:**
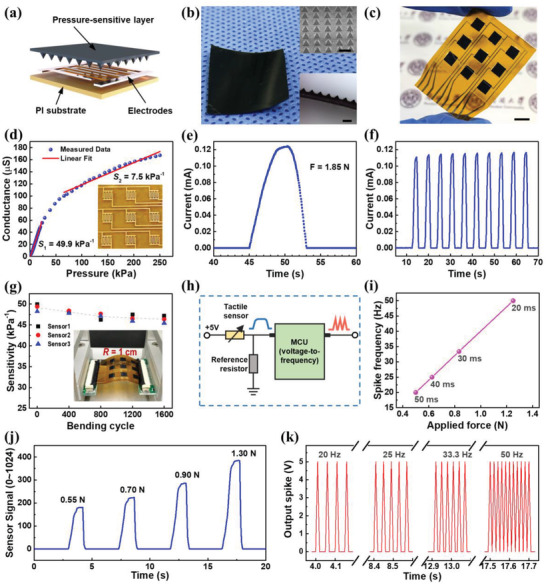
Performance of the flexible tactile sensor. a) Schematic illustration of sensor structure. b) Photograph of pressure‐sensitive elastic layer made of carbon nanotube and room‐temperature‐vulcanizing latex. Inset: optical images showing arrangement and cross‐section of micropyramid arrays on the surface of this layer. c) Photograph of flexible tactile sensor with 3 × 3 sensing elements. d) Relationship between applied pressure and sensor conductance. Applied pressure of 250 kPa corresponds to applied force of 25 N. Inset: photograph of interdigitated electrodes of the sensor. e) Time‐dependent response of the sensor under slowly‐applied force and f) under rapidly applied force. g) Sensitivity of the sensor under repeated bending over 1600 cycles. Inset: photograph of the sensor under bending. h) Schematic circuit diagram of the connection between sensor and spike‐encoding circuit. i) Relation between applied force and output spike frequency, showing the spike‐rate coding strategy. j) Sensor signal and k) output spike recorded under different applied force. The scale bar in (b) and (c) are 100 µm and 5 mm.

The tactile sensor was constructed by laminating the pressure‐sensitive layer on a polyimide film coated with gold electrodes (**Figure** **3**a). The conductive sensing layer bridges the interdigitated electrodes. The pressure‐sensitive layer is made of a composite of carbon nanotubes (CNTs) and room‐temperature‐vulcanizing (RTV) latex, and its surface is uniformly patterned with pyramidal microstructures (Figure 3b). Compared with other pressure‐sensitive material such as CNT/PDMS composite,^[^ ^32^, ^33^ ^]^ the CNT/RTV composite is easy to handle and process, because its precursor dissolved in water has lower viscosity (≈3.5 cP) than PDMS precursor and also its curing time (<30 min) is shorter than that of PDMS. A sensor array (Figure 3c) was fabricated by mechanically cutting the pressure‐sensitive layer to small pieces of sensing elements, and then encapsulating them using adhesive medical dressing. The sensor array can be directly connected to other circuit by a flexible printed circuit (FPC) connector. Conductance of the sensor under different pressures was measured, and the sensitivities of the sensor are 49.9 and 7.5 kPa^−1^ at low‐pressure range (0–20 kPa) and high‐pressure range (65–250 kPa), respectively (Figure 3d). The pressure response of the sensor is quite linear in the low‐pressure range, which is confirmed by the high correlation coefficient (*R* = 0.9981) of linear fit (Figure S4, Supporting Information). The performance of the sensor is comparable to those of other resistive tactile sensors reported elsewhere (Table S1, Supporting Information).^[^ ^3^, ^14^, ^32^ ^]^ Time‐resolved current output of the sensor under constant applied voltage exhibits good static response and high dynamic stability upon the stimulation of slowly applied force (Figure 3e) and rapidly applied force (Figure 3f). The sensitivity of the sensor shows no obvious degradation during cyclic bending test (Figure 3g), proving its high mechanical resilience and bending stability. The linear response and mechanical stability of this sensor can be attributed to the stress concentration of the micropyramid tips and the mechanical flexibility of the sensing material, so the sensor is suitable for use in robotic manipulation and tactile interaction.

A spike‐encoding circuit using a microcontroller was designed to convert the sensor signal to presynaptic spikes (Figure 3h). Spike‐rate coding is one of the neural coding strategies used by single neurons, in which the reciprocal of a single interspike interval (instantaneous rate) correlates with the stimulus attribute (e.g., amplitude).^[^ ^5^, ^28^, ^34^ ^]^ In addition, receptor potentials generated by sensory receptors have a threshold of stimulus amplitude which must be reached before a response is generated.^[^ ^1^, ^5^ ^]^ Herein, a spike‐rate coding scheme was implemented to generate spikes (constant amplitude and duration: 5 V, 10 ms) with frequency (20–50 Hz) linear to the applied force (0.50–1.25 N), and a thresholding value of 0.5 N was introduced (Figure 3i). The spike‐encoding scheme was examined by simultaneously measuring the sensor signal (Figure 3j) and the output spike (Figure 3k) under different applied forces. The applied forces with different amplitudes were effectively transformed to spikes with various frequency, which can be used as presynaptic spikes to stimulate the synaptic device.

### 2.4 Material Hardness Classification

**Figure 4:**
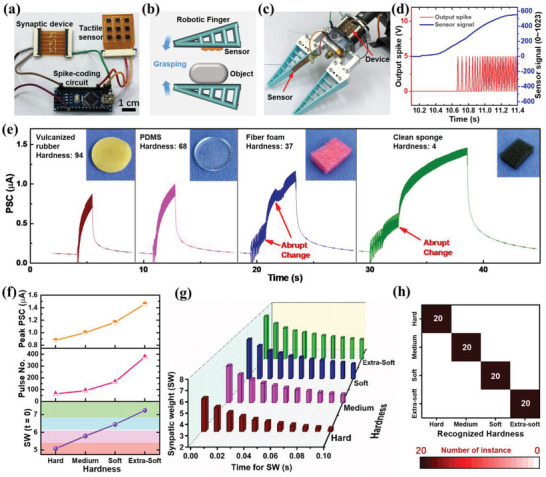
Neuromorphic classification of material hardness during robotic grasping. a) Photograph of flexible artificial sensory nerve. b) Schematic illustration and c) experimental setup of material‐hardness identification. A two‐finger robotic gripper equipped with the artificial sensory system was closing at constant speed to grasp a target material. d) Typical response of the sensor and corresponding spike trains generated from the spike‐coding circuit, when the robotic gripper was grasping a soft object. e) PSC response of the synaptic device when vulcanized rubber, PDMS, fiber foam, and cleaning sponge were tested. Insets: photographs of the materials. f) Relationship between material hardness and device output (peak PSC, pulse number and SW). SW alone can be used for hardness classification. g) SW recorded at different times after release of tactile stimuli, showing that hardness information was stored in the device as sensory memory. h) Classification results of material hardness by reading the device output. In the confusion matrix, each row and each column represent the actual and recognized hardness respectively, and thus the diagonal elements correspond to the correct classifications.

Hardness is among the most important attributes of an object that humans learn about through touch.^[^ ^2^ ^]^ Humans are extremely proficient in recognizing surface properties and discerning physical properties of unknown objects by performing tactile exploration even without visual feedback.^[^ ^1^, ^6^ ^]^ Advanced robots that have tactile recognition capabilities could increase the precision of dexterous manipulation, and the safety of such operations.^[^ ^10^ ^]^ Here, real‐time identification of material hardness was achieved by using the flexible artificial sensory nerve. This system was fabricated in a compact and portable form (**Figure** **4**a), and therefore it can be readily integrated with robotic systems. Hardness identification was performed by using a two‐finger robotic gripper to slowly grasp and quickly release a target material. The contact force between finger and object during such active touch was detected by the tactile sensor and then processed by the synaptic device, which were attached on the robotic finger (Figure 4b,c).

The response of the sensor and the output of the spike‐encoding circuit were examined simultaneously at first. The sensor signal that corresponds to the increasing grasping force was effectively converted to frequency‐encoded spike trains with thresholding (Figure 4d). Four materials (vulcanized rubber, PDMS, fiber foam, cleaning sponge) with decreasing hardness were tested, and during the process of grasping and releasing, the PSC was recorded in real time (Figure 4e). The PSC value increases gradually during the grasping process. Given that the maximum grasping force was fixed in the robotic gripper, the duration of the grasping process decreases as the hardness of the material increases (Table S2, Supporting Information). During grasping of porous materials with low hardness, the PSC shows some abrupt and inconsistent changes, which is probably related to the release of compressive strain in the material. The PSC is essentially the temporal integration of the stimuli,^[^ ^35^ ^]^ so the profile of the PSC reflects the grasping process in the hardness test.

To quantitatively evaluate the material hardness, three different metrics including peak PSC, pulse number, and synaptic weight were extracted from the PSC curves. All the three metrics can discern the material hardness, as manifested by the changes in their values (Figure 4f). Among these three metrics, the synaptic weight is most suitable as a classification criterion to differentiate the material hardness. The synaptic weight that corresponds to the four materials (synaptic weight: 5.1, 5.8, 6.4, 7.2) increases as the material hardness decreases (Shore‐00 hardness: 94, 68, 37, 4), and interestingly, a nearly linear relationship between them was observed (Figure S5, Supporting Information). Note that Shore‐00 hardness scale ranges from 0 to 100, corresponding to extra‐soft and hard materials.^[^ ^36^ ^]^ The synaptic weight obtained after releasing the grasping force (Figure 4g) remains distinct among the four cases after the tactile stimuli stops, indicating that the hardness information can be stored in the device as short‐time sensory memory (up to a few hundreds of milliseconds). To better evaluate the performance of hardness discrimination, two PDMS samples (Shore‐00 hardness: 63 and 61) with similar hardness were tested (Figure S6, Table S3, Supporting Information), and the results demonstrate that our system can effectively differentiate material hardness as small as 2.

For hardness classification, the decision boundary was simply chosen at the midpoint of the line that connects two adjacent synaptic weights (Figure 4f). Accordingly, the four materials with different hardness can be classified by reference to synaptic weight (SW for hard material: 4.7–5.4; SW for medium‐hard material: 5.4–6.1; SW for soft material: 6.1–6.8; SW for extrasoft material: 6.8–7.5). The classification results of material hardness, represented as a confusion matrix (Figure 4h), demonstrate that the hardnesses of the materials were all identified correctly. This classification task was performed by reading the device output without using algorithms or computing resources. Our system is more advantageous for hardness discrimination compared with previously reported works (Table S4, Supporting Information), since our system adopts neural coding and synaptic processing of tactile signal without using additional instrument or computer, and achieves accurate discrimination of material hardness in a wide range.

### 2.5 Tactile Pattern Recognition

**Figure 5:**
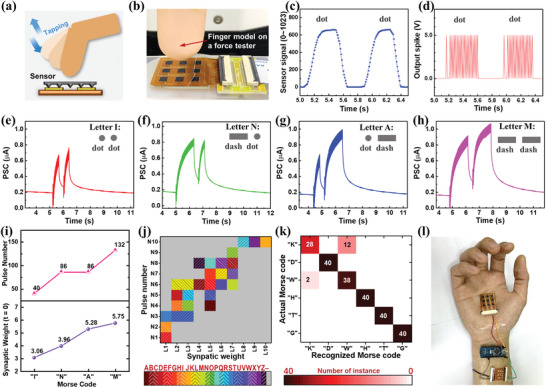
Neuromorphic recognition of Morse‐code tactile patterns during tactile interaction. a) Schematic illustration and b) experimental setup of generating Morse‐code tapping patterns. c) Typical response of the sensor and d) corresponding spike train when two “dots” are consecutively tapped. e–h) PSC response of the synaptic device when Morse code for (e) letter “I”, (f) letter “N”, (g) letter “A”, and (h) letter “M” were tapped. i) Relationship between Morse code and device output (synaptic weight and pulse number). j) Color mapping of the 26 letters from “A” to “Z” for recognizing Morse code using synaptic weight and pulse number. Synaptic weight and pulse number are both divided into ten ranges, and this mapping considers the variation of device output by running each tests for three times. k) Recognition results of Morse‐code tapping patterns by reading the device output. In the confusion matrix, the off‐diagonal element indicates that the system has difficulties in distinguishing categories, while diagonal element represents correct recognition. l) Photograph of flexible artificial sensory nerve attached to a human hand.

Tactile interaction is useful for communication and human–robot interfaces, and humans rely on the sensory memory to process sensory information and further achieve the perception and recognition of tactile stimulation and patterns.^[^ ^9^, ^18^, ^34^ ^]^ Recognition of tactile patterns was implemented in our system by tapping Morse code, which were generated by manually control the tapping movement of a finger model mounted on a force‐feedback test stand (**Figure** **5**a,b). The Morse code is composed of dash (**–**) and dot (**∙**) that are represented respectively by long and short contact times during a tap. In telegraph communication, the duration of dash, dot, and interval are set as 3:1:1, respectively.

The spike encoding function was first tested by tapping two dots, and the sensor signal (Figure 5c) in response to this tactile event was effectively converted to frequency‐encoded spikes (Figure 5d) with durations of ≈10 ms separated by intervals of ≈10 ms. The interval between two taps is comparable to the time constant of the memory loss curve of the synaptic device, and the device can retain the sensory memory during the tapping interval. Then Morse codes of letters I (**∙ ∙**), N (**– ∙**), A (**∙ –**), or M (**– –**) were tapped as tactile input, while PSC from the synaptic device was recorded in real time. The four letters produce PSC curves that have distinct profiles (Figure 5e–h). Letter M and letter I lead to largest and smallest PSC, respectively, because contact time of tapping is the longest for M and the shortest for I. The contact times of tapping for letter N and letter A are nearly the same, but A (**∙ –**) yields a larger PSC after tactile event than N (**– ∙**). This may be due to the memory loss effect of the first tap and the facilitation effect of the second.

Two important metrics including pulse number and synaptic weight extracted from the PSC curve were plotted as device output with respect to the input of Morse code (Figure 5i). The synaptic weight can well distinguish the four Morse code letters. Due to the rapid decay of the sensory memory in our synaptic device, the influence of the first tap on the synaptic weight is not strong, as manifested by the slight difference in synaptic weight between letter A (**∙ –**) and letter M (**– –**). Therefore, to improve the classification accuracy, both pulse number and synaptic weight were adopted as classification criteria for Morse code recognition. This use of pulse number for sensory information analysis and processing is reasonable, because in neuroscience, spike counts of neurons are measured in a similar way over the timescale of a stimulus presentation or a behavior trail (typically hundreds of milliseconds), and spike count correlations among multiple neurons are used to study cortical processing and neuron connectivity.^[^ ^37^ ^]^

Further tests were performed by tapping the Morse codes of the alphabet, while recording the response of the system in real time. An interval of 5–6 s between letters was set to ensure that the quiescent current settled to the current level of ≈0.2 µA, so the system could operate in a continuous mode without the need to apply inhibitive presynaptic pulses to reset the PSC between different tactile events. Three runs of test were performed, and the decision boundaries for pulse number and synaptic weight were set appropriately (Table S5, Supporting Information) to achieve the classification of 26 letters on a 10 × 10 mapping (Figure 5j). As a practical demonstration of tactile recognition, six random letters were input and identified by matching the device output to the 2D mapping. The classification results represented as a confusion matrix (Figure 5k) reveal that the recognition accuracy is 0.94, which is comparable to those of other synaptic tactile sensory systems designed for tactile recognition tasks (Table S6, Supporting Information). When letters have similar and long Morse‐code patterns, the error rate increases, probably due to the decay of sensory memory during multiple taps. Given that our system can be directly attached to human body in wearable form (Figure 5l), this demonstration of the capability to distinguish Morse codes implies that our system has the potential to be used for smart interfaces and human–robot interaction.

Our system demonstrates several advantages over other artificial tactile sensory systems reported in literature (Table S7, Supporting Information). First, neuromorphic tactile recognition was directly implemented at the device level by utilizing the intrinsic characteristics of the synaptic device without relying on algorithm or computing resource, and thus enabling the applications in robotic manipulation and smart interfaces which require real‐time perception and energy‐efficient recognition. Second, the tactile event was detected in continuous mode without applying erasing signals or inhibitive spikes to reset the synaptic device, and therefore our system can achieve continuous and sustained operation. Third, the tactile‐sensing and sensory‐processing components in our system were prepared on flexible substrates using low‐cost fabrication, and notably the synaptic device was fabricated using a facile and scalable method of interfacial self‐assembly, so the system is suitable to be used as wearable/flexible electronics.^[^ ^23^, ^38^ ^]^ To the best of our knowledge, this is the first work that exploits nanoparticle self‐assembly method to construct flexible synaptic devices. The self‐assembly fabrication approach does not require high‐temperature processing or post‐treatment, and can be extended to a broad range of semiconducting nanocrystals. Besides, the synaptic device built from self‐assembled nanoparticles shows excellent performance of mechanical flexibility, synaptic facilitation, and sensory memory (Table S8, Supporting Information), which can be ascribed to the fact that the semiconducting film of self‐assembled nanoparticles is uniform, continuous, and defect‐less, and hence it is favorable for charge carrier transport and proton gating. The electronic properties of the self‐assembled nanoparticles can be further tuned by tailoring the morphology, composition, and surface chemistry of the nanocrystals.^[^ ^39^ ^]^ Moreover, self‐assembly fabrication achieves the bottom‐up preparation of synaptic device at flexible substrates without using vacuum deposition, enabling potential applications in wearable electronics. Our future work includes miniaturization and optimization of the spike encoding circuit (by adding reading function of PSC) to form hybrid flexible electronics, and incorporation of tunable synaptic plasticity and long‐term memory in the synaptic device to realize adaptation and learning of tactile stimuli and tactile patterns.

## 3 Conclusion

In summary, a flexible artificial sensory nerve for neuromorphic tactile recognition is developed. It emulates the neural coding and neural processing of tactile information in human sensory nerve. By integrating the flexible tactile sensor, the flexible synaptic device and the spike‐encoding circuit, this system is built as a mimicry of a biological somatosensory system that is composed of sensory receptors, sensory neurons, and synapses. Notably, the channel of the synaptic transistor is fabricated as a structurally continuous and mechanically robust film of semiconductor nanoparticles using an interfacial self‐assembly technique, which allows large‐scale and facile transfer to flexible substrates. The time constants of the neural facilitation and sensory memory of the synaptic device are comparable to those of biological counterparts. By using device output including pulse number and synaptic weight as classification criteria, the system achieves identification of material hardness during robotic grasping and recognition of Morse‐code tapping patterns during tactile interaction. The experimental demonstrations show that tactile recognition was realized in a real‐time and near‐sensor manner without using computing resource or algorithm. Our system simplifies the fabrication process and also expands the recognition capabilities of neuromorphic sensory systems, and it may find applications in intelligent robots, human–machine interfaces, and smart wearable devices, which require real‐time perception and efficient recognition.

## 4 Experimental Section

### Nanoparticle Self‐Assembly

Commercially available ZnO NPs with small size (10–15 nm, 2.5 wt% in isopropanol, Sigma‐Aldrich) and large size (≈50 nm, 40 wt% in isopropanol, Aladdin) are both suitable for self‐assembly. Surface morphology comparison of the self‐assembled NP films prepared using these two types of NPs are presented in the AFM images in Figure S7 of the Supporting Information. Typically, the small ZnO NPs were used in the self‐assembly experiment. The NP dispersion was ultrasonicated for 10 min and then centrifuged at 2000 × *g* to discard the precipitated material. Then, Milli‐Q water was filled into a glass petri dish (7 cm diameter), and the NP dispersion (15 µL) was gently dropped using a micropipette onto the water surface from a height of 1 cm, and then the petri dish was covered using a glass lid. Consequently, a clearly visible continuous membrane formed at the air–water interface. After tens of seconds, this floating film was slowly transferred to a desired substrate and then allowed to dry for 10 min.

### Fabrication of Synaptic Device

A polyimide substrate was ultrasonically cleaned in acetone, isopropanol, and deionized water, sequentially. Source, drain, and in‐plane gate were then thermally deposited through a shadow mask, which defines a channel length of 500 µm. The substrate was treated with UV‐ozone for 10 min, and then the self‐assembled NP film was transferred onto the substrate surface to produce the channel. Facile heat treatment (100 °C, 30 min) was performed to eliminate remaining water and improve the contact between NPs and electrodes. Ion‐gel electrolyte was obtained by drying chitosan solution deposited on glass slide (80 °C, 15 min). The solution was prepared by mixing chitosan (Sigma‐Aldrich), acetic acid (TCI), and deionized water in a weight ratio of 1:1:50 at room temperature. A small amount of glycerol (≈1 wt%) was added to the chitosan solution to promote formation of ion gel. The ion gel electrolyte was used to cover the channel and the gate. Also, a final elastomer layer of styrene–ethylene–butylene–styrene can be used for device encapsulation.

### Fabrication of Tactile Sensor

A solution of multiwalled carbon nanotubes (MWCNTs, 10 µm length, 14 wt% in water, XF Nano) was mixed with RTV latex (40 wt% in water, Lida Latex) in a volume ratio of 1:2. The dark gray mixture was spin‐coated (1000 rpm, 20 s) on a silicon mold etched with inverse pyramidal structures (size 60 µm, pitch 100 µm). The CNT/RTV composite film was dried for 30 min, and then peeled from the silicon mold and mechanically cut into 3 mm × 3 mm pieces to produce the sensing elements. A polyimide film was rinsed with acetone, isopropanol, and deionized water, and then patterned with interdigitated electrodes (Ti/Au, 5 nm/100 nm). The sensing elements were placed on top of the interdigitated electrode region of the polyimide film, and then a Tegaderm film (FDA‐approved biocompatible adhesive dressing, 3M) was laminated on top of the sensor under applied pressure of 1.2 kPa for 30 min.

### System Integration

The flexible substrate and the electrode design allow both the flexible tactile sensor and the flexible synaptic transistor to be directly fitted into an FPC connector (20 pins, 1 mm pitch) and further connected to a spike‐encoding circuit to form a sensory system. In a typical setup, the signal of the tactile sensor was sent to the analog input of a microcontroller (ATmega328P, Atmel) via a voltage‐divider circuit, while the gate of the synaptic transistor was driving by the output of the microcontroller in the form of frequency‐coded spikes. The microcontroller serves as the spike‐encoding circuit, and transforms the sensor signal (in the range of 0 to 1023 corresponding to 0–5 V) to frequency‐coded spikes (amplitude of 5 V) at a sampling rate of 100 Hz. The function of microcontroller is similar to a voltage‐controlled oscillator. The coding strategy of output spike is derived from the Izhikevich model of cortical spiking neurons, in which the spike rate is proportional to the applied pressure. Electrical responses of the sensory system were evaluated by real‐time measurement of the current output of the synaptic device during application of pressure to the tactile sensor, with a common ground configuration of the microcontroller and the measurement instrument.

### Characterization and Measurement

Optical images were obtained using a Leica microscope. TEM images were taken by a JEOL JEM‐2100F electron microscope. SEM images were taken by a Thermo Scientific Apreo‐S field‐emission SEM. AFM images were acquired using a Bruker dimension icon AFM operated in tapping mode. XRD patterns were recorded by a Rigaku Ultima‐IV X‐ray diffractometer. Electrical measurements of the synaptic device were performed using a Keithley 4200A semiconductor parameter analyzer and a probe station in ambient environment at room temperature. Pressure‐dependent electrical measurements of the tactile sensor were performed using a Keithley 2400 source meter and a Mark‐10 force test system, which includes a motorized test stand and a force gauge. Bending test was performed by mounting the sensor or the device on a Prtronix flexible electronics bending tester via FPC connectors (Figure S8, Supporting Information). Hardness classification was performed using a two‐finger robotic gripper that can execute grasping and releasing operations. Four common materials including vulcanized rubber (used in pneumatic tires), PDMS (base/curing agent ratio 20:1), fiber foam (used in packaging), and cleaning sponge (used in household cleaning) were tested. The Shore‐00 hardness of these materials was measured using a Rex MSDD‐4 multiscale digital durometer. Identification of Morse‐code tapping patterns was conducted by manually operating a force gauge test stand and controlling its movement (up and down).

### The Statement for Human Subjects

A disclaimer of signed informed consent from the person who participated in the experiments with human subject was obtained.

## Conflict of Interest

The authors declare no conflict of interest.

## Supporting information

Supporting Information

## Data Availability

The data that support the findings of this study are available in the supplementary material of this article.
