# Supplementary material for: A Flexible Artificial Sensory Nerve Enabled by Nanoparticle‐Assembled Synaptic Devices for Neuromorphic Tactile Recognition
Source: Adv Sci (Weinh). 2022 Jun 9;9(24):2106124. doi: 10.1002/advs.202106124 (PMC9405521; doi:10.1002/advs.202106124)
Supplement: Supplementary file 1 — Supporting Information [file ADVS-9-2106124-s001.pdf]

ADVANCED SCIENCE
Open Access

Supporting Information

for *Adv. Sci.*, DOI 10.1002/advs.202106124

A Flexible Artificial Sensory Nerve Enabled by Nanoparticle-Assembled Synaptic Devices for Neuromorphic Tactile Recognition

*Chengpeng Jiang, Jiaqi Liu, Lu Yang, Jiangdong Gong, Huanhuan Wei and Wentao Xu**

Supporting Information

A Flexible Artificial Sensory Nerve Enabled by Nanoparticle-Assembled Synaptic Devices for Neuromorphic Tactile Recognition

*Chengpeng Jiang, Jiaqi Liu, Lu Yang, Jiangdong Gong, Huanhuan Wei, Wentao Xu**

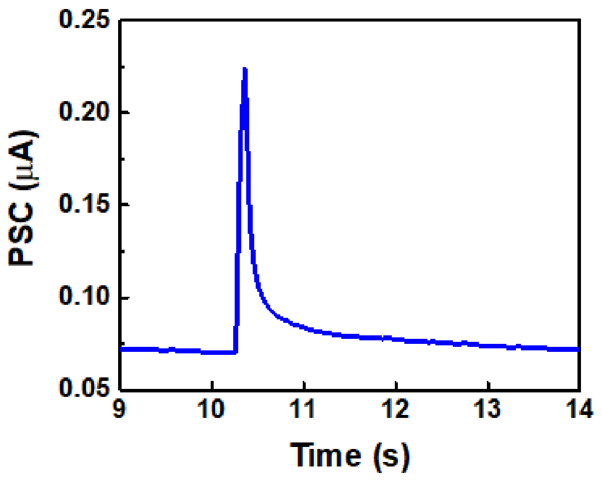

Figure S1. Excitatory postsynaptic current (EPSC) of the flexible synaptic device in response to a single spike (5 V, 50 ms) under bias voltage V_{ds} = 1 V.

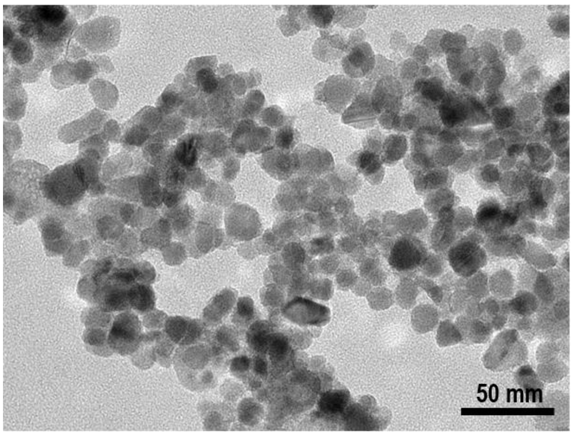

Figure S2. TEM image of the ZnO NPs used for fabricating the synaptic device.

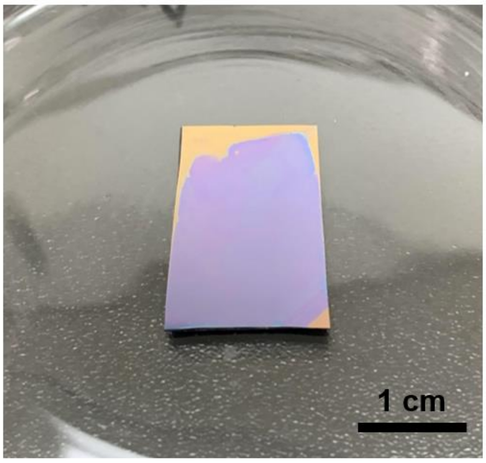

Figure S3. Photograph of a Si/SiO_2 substrate with self-assembled NPs on its surface.

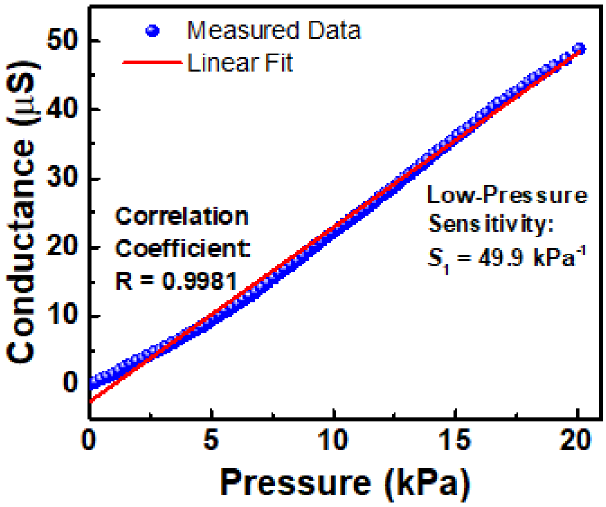

Figure S4. Relationship between applied pressure and sensor conductance at low-pressure range (0–20 kPa), in which applied pressure of 20 kPa corresponds to applied force of 2 N. Correlation coefficient *R* of the linear fit is 0.9981, and sensitivity of the sensor is 49.9 kPa^{-1}.

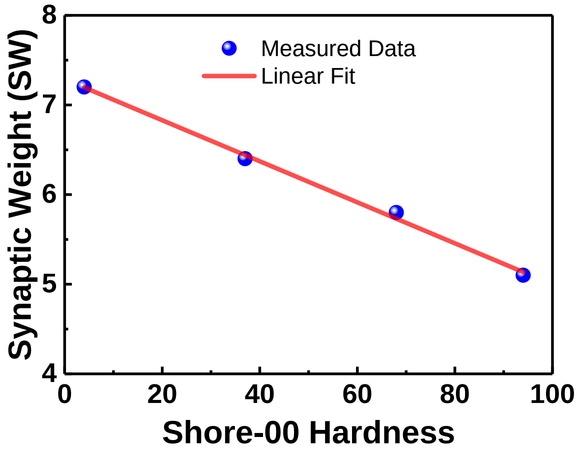

Figure S5. Relationship between the material hardness (in Shore-00 scale) and the synaptic weight of the synaptic device.

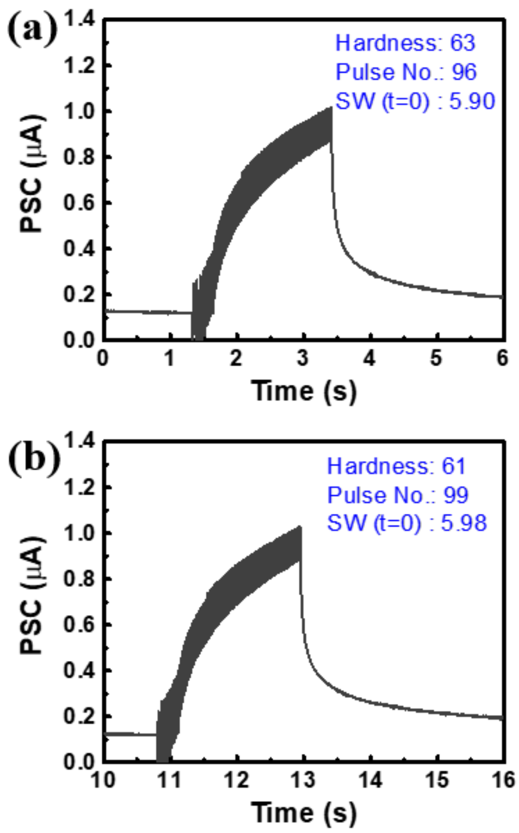

Figure S6. Postsynaptic current (PSC) response of the synaptic device when two PDMS samples with similar hardness were tested for hardness discrimination. (a) PSC response for PDMS with Shore hardness of 63. (b) PSC response for PDMS with Shore hardness of 61.

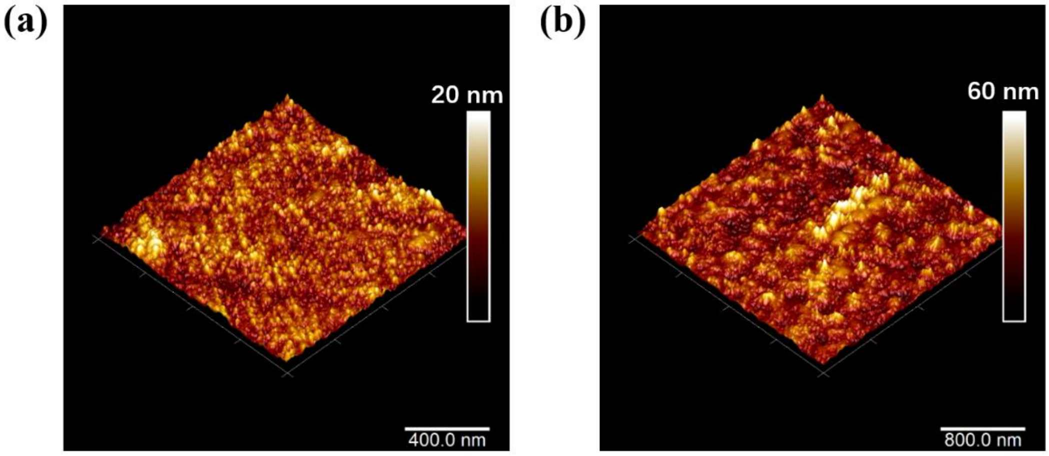

Figure S7. AFM characterization of the ZnO NP films fabricated using an interfacial self-assembly technique. (a) AFM image of the sample prepared using small NPs (diameter ~10 nm). (b) AFM image of the sample prepared using large NPs (diameter ~50 nm).

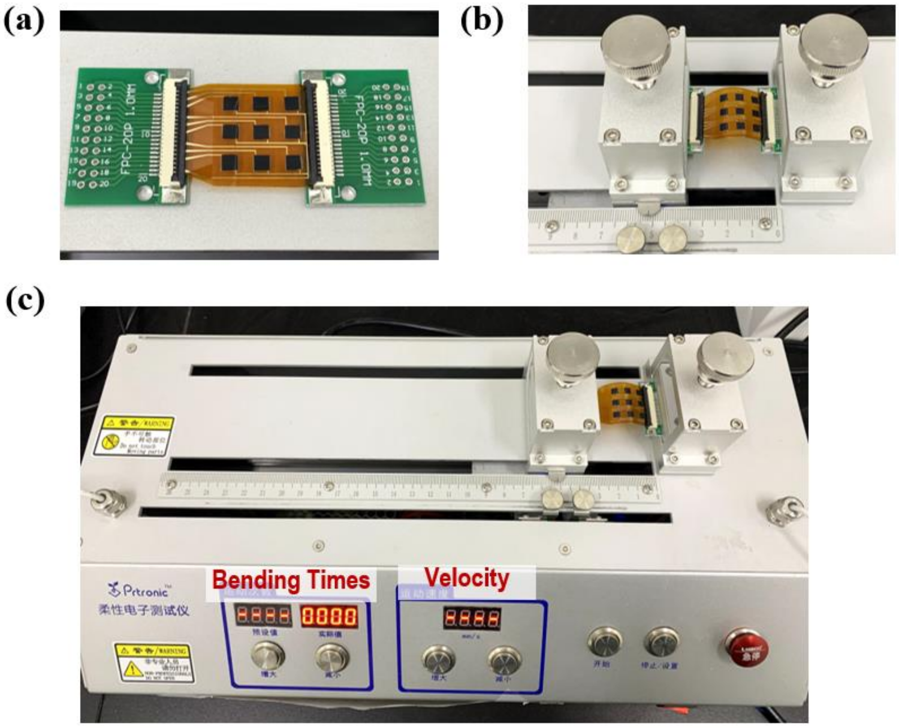

Figure S8. Bending test setup for flexible electronics. (a) The sensor or the device is fixed by two FPC connectors on two ends. (b) The sensor or the device is mounted on a flexible electronics bending tester. (c) Mechanical bending with pre-defined bending radius (in our case, R = 1 cm) is applied by controlling the experimental parameters of the equipment.

Table S1. Comparison of recent works on resistive tactile sensors.

Materials	Sensitivity*	Maximum Pressure (Detection range)	Reference
CNTs/PDMS	65 kPa^{-1}	1 kPa	Adv. Mater. 30, 1801291 (2018)[S1]
CNTs/leather	32 kPa^{-1}	1 kPa	Adv. Sci. 6, 1801283 (2019)[S2]
MXene/PDMS	54 kPa^{-1}	100 kPa	Nat. Commun. 11, 1369 (2020)[S3]
Graphene/PDMS	8.5 kPa^{-1}	12 kPa	Adv. Mater. 28, 5300 (2016)[S4]
CNTs/cotton fabric	14.4 kPa^{-1}	15 kPa	Adv. Mater. 29, 1703700 (2017)[S5]
CNTs/graphene/PDMS	19.8 kPa^{-1}	6 kPa	Adv. Funct. Mater. 27, 1606066 (2017)[S6]
CNTs/RTV latex	49.9 kPa^{-1}	250 kPa	This work

* Because tactile sensors exhibit different sensitivities at different pressure ranges, the collected data shows the highest sensitivity reported in each literature

Table S2. Relationship between the hardness of the materials and the duration of the grasping process.

**Material**	Vulcanized rubber	PDMS	Fiber foam	Cleaning sponge
**Hardness Shore-00**	94	68	37	4
**Duration of Grasping Process**	1.3 s	2.0 s	4.0 s	8.6 s

Table S3. Discrimination of materials with similar hardness. Two PDMS samples with similar hardness (Shore 00: 63 and 61) were tested, and the device output including pulse number and synaptic weight are compared.

Material Hardness	Pulse Number	Synaptic Weight
63	96	5.90
61	99	5.98

Table S4. Comparison between different tactile sensory systems for hardness discrimination tasks. Hardness values are converted to Shore-00 scale for clarity.

Type of Tactile Sensory System	Form of Tactile Signal	Processing of Tactile Signal	Hardness Discrimination Performance	Discern Extra-soft Materials	Reference
Fiber-optic mechanoreceptor	Optical signal	Optical detector, computer	Hardness range: 45 – 100 Hardness difference: 10	No	IEEE Sens. J. 17, 5123 (2017)[S7]
Optical micro/nanofiber tactile sensor	Optical signal	Spectrometer, computer	Hardness range: 73 – 91 Hardness difference: 4	No	ACS Appl. Mater. Interfaces 13, 4560 (2021)[S8]
Deep learning based GelSight* tactile sensor	Tactile images	Video recorder, computer running ML algorithm	Hardness range: 8 – 87 Hardness difference: ~5	Yes	IEEE ICRA 7989116 (2017)[S9]
Triboelectric nanogenerator dual-mode tactile sensor	Triboelectric signal	Electrometer, DAQ system	Material: PDMS, copper, terylene, glass	No	ACS nano 11, 3950 (2017)[S10]
Tactile Avatar System	Piezoelectric signal	Oscilloscope, DAQ system, computer running ML algorithm	Hardness range: 20 – 90 Hardness difference: 10	No	Adv. Sci. 8, 2002362 (2021)[S11]
Flexible artificial sensory nerve	Spike trains	Built-in synaptic device	Hardness range: 4 – 94 Hardness difference: 2	Yes (including porous material)	This work

* GelSight is a commercially available device that is used as a scanner for acquiring surface texture and shape, and it consists of a slab of clear elastomer covered with a reflective skin.

Table S5. Decision boundaries for the pulse number and the synaptic weight. The names of decision boundaries (N1–N10, L1–L10) correspond to the notations in Figure 5j.

Decision boundary	Pulse number (range)
N1	10~30
N2	30~50
N3	50~70
N4	70~90
N5	90~120
N6	120~140
N7	140~160
N8	160~190
N9	190~210
N10	210~230

Decision boundary	Synaptic weight (range)
L1	2.7~3.4
L2	3.4~3.8
L3	3.8~4.3
L4	4.3~4.9
L5	4.9~5.4
L6	5.4~5.6
L7	5.6~6.0
L8	6.0~6.4
L9	6.4~6.7
L10	6.7~7.1

Table S6. Comparison between different tactile sensory systems designed for tactile pattern recognition tasks.

Tactile Sensory System	Tactile Recognition Task	Accuracy	Notes	Reference
Tactile ferroelectric skin	Handwriting pattern recognition	82% – 99%	Training time 10	Adv. Sci. 7, 2001662 (2020)[S12]
Synaptic device with tactile memory	Tactile image recognition	92.5%	Training time >100	Nano Energy 76, 105109 (2020)[S13]
Artificial tactile sensor system	Tactile pattern recognition	93%	Training time 50	NPG Asia Mater. 12, 76 (2020)[S14]
Flexible artificial sensory nerve	Morse-code tactile pattern recognition	94%	/	This work

Table S7. Comparison between different artificial tactile sensory systems in terms fabrication, flexibility, implementation and functions.

**Type of artificial tactile sensory system**	**Facile and low-cost fabrication (Yes / No)**	**Flexible form (Yes / No)**	**Tactile recognition at device level (Yes / No)**	**Tactile recognition functions**	**Reference number**
Neuromorphic tactile processing system	Yes	Yes (both sensor and synaptic device)	No (algorithm required)	Braille code recognition	Ref. 14 Adv. Mater. 30, 1801291 (2018).
Artificial neural tactile sensing system	Yes (only for sensor)	No	No (processing hardware required)	Surface texture recognition	Ref. 16 Nat. Electron. 4, 429-438 (2021).
Neuro-inspired artificial peripheral nervous system	Yes (only for sensor)	No	No (processing hardware and algorithm required)	Grating pitch classification; object shape classification	Ref. 7b Sci. Robot. 4, eaax2198 (2019).
Bioinspired optoelectronic spiking afferent nerves	Yes (only for sensor)	No	No (algorithm required)	Handwriting classification; braille recognition	Ref. 3 Nat. Commun. 11, 1-9 (2020).
Bioinspired flexible organic artificial afferent nerve	Yes (only for sensor)	Yes (both sensor and synaptic device)	No (frequency analysis required)	Movement recognition; braille recognition	Ref. 13 Science 360, 998-1003 (2018).
Flexible artificial tactile sensory nerve	Yes	Yes (both sensor and synaptic device)	Yes	Material hardness identification; Morse-code tactile pattern recognition	*This work

Table S8. Comparison between different synaptic devices in terms of fabrication, flexibility, bending stability, signal processing functions, and synaptic functions.

Synaptic Transistor	Fabrication Method	Flexibility	Synaptic Functions*	Applications	Reference
Stretchable synaptic transistor	Inkjet print (purified swCNT)	High (stretchable)	SNDP, SRDP, SDDP	Skin electronics (not demonstrated)	Nat. Commun. 10, 2676 (2019)[S15]
Heterojunction synaptic device	Hydrothermal synthesis, magnetron sputtering (MoS_2 / TiN_xO_{2-x})	No	SNDP, SRDP, SDDP, STDP, Long/short term memory, PPF 136%	Visual perception and memory	Adv. Funct. Mater. 31, 2101201 (2021)[S16]
Contact electrification activated synaptic transistor	CVD deposition (MoS_2)	No	SNDP, SDDP, sensory memory, PPF 140%,	Spatiotemporal tactile sensing	Nat. Commun. 12, 1581 (2021)[S17]
Multi-terminal neuro-transistor	Magnetron sputtering (IGZO)	No	SNDP, SRDP, SDDP, short term memory, PPF 250%	Spatiotemporal sound detection	Adv. Mater. 31, 1900903 (2019)[S18]
Flexible ferroelectric organic FET	Thermal evaporation (Pentacene)	High (bending $>10^4$)	SNDP, SRDP, SDDP, short term memory, PPF 108%	Tactile memory	Nat. Commun. 11, 2753 (2020)[S19]
Self-assembled flexible synaptic transistor	Self-assembly (ZnO)	High (bending $>10^3$)	SNDP, SRDP, SDDP, sensory memory, PPF 218%	Tactile classification and recognition	This work

* Abbreviations: spike number dependent plasticity (SNDP), spike rate dependent plasticity (SRDP), spike duration dependent plasticity (SDDP), and spike timing dependent plasticity (STDP).

Supplementary References:

[S1] C. Wan, G. Chen, Y. Fu, M. Wang, N. Matsuhisa, S. Pan, L. Pan, H. Yang, Q. Wan, L. Zhu, *Advanced Materials* 2018, 30, 1801291.

[S2] B. Zou, Y. Chen, Y. Liu, R. Xie, Q. Du, T. Zhang, Y. Shen, B. Zheng, S. Li, J. Wu, *Advanced Science* 2019, 6, 1801283.

[S3] H. Tan, Q. Tao, I. Pande, S. Majumdar, F. Liu, Y. Zhou, P. O. Persson, J. Rosen, S. van Dijken, *Nature Communications* 2020, 11, 1369.

[S4] G. Y. Bae, S. W. Pak, D. Kim, G. Lee, D. H. Kim, Y. Chung, K. Cho, *Advanced Materials* 2016, 28, 5300.

[S5] M. Liu, X. Pu, C. Jiang, T. Liu, X. Huang, L. Chen, C. Du, J. Sun, W. Hu, Z. L. Wang, *Advanced Materials* 2017, 29, 1703700.

[S6] M. Jian, K. Xia, Q. Wang, Z. Yin, H. Wang, C. Wang, H. Xie, M. Zhang, Y. Zhang, *Advanced Functional Materials* 2017, 27, 1606066.

[S7] M. Fajkus, J. Nedoma, R. Martinek, V. Vasinek, H. Nazeran, P. Siska, *Sensors* 2017, 17, 111.

[S8] Y. Tang, H. Liu, J. Pan, Z. Zhang, Y. Xu, N. Yao, L. Zhang, L. Tong, *ACS Applied Materials & Interfaces* 2021, 13, 4560.

[S9] W. Yuan, C. Zhu, A. Owens, M. A. Srinivasan, E. H. Adelson, "Shape-independent hardness estimation using deep learning and a gelsight tactile sensor", presented at *2017 IEEE International Conference on Robotics and Automation (ICRA),* 2017.

[S10] T. Li, J. Zou, F. Xing, M. Zhang, X. Cao, N. Wang, Z. L. Wang, *ACS nano* 2017, 11, 3950.

[S11] K. Kim, M. Sim, S. H. Lim, D. Kim, D. Lee, K. Shin, C. Moon, J. W. Choi, J. E. Jang, *Advanced Science* 2021, 8, 2002362.

[S12] K. Lee, S. Jang, K. L. Kim, M. Koo, C. Park, S. Lee, J. Lee, G. Wang, C. Park, *Advanced Science* 2020, 7, 2001662.

[S13] D. Wang, L. Wang, W. Ran, S. Zhao, R. Yin, Y. Yan, K. Jiang, Z. Lou, G. Shen, *Nano Energy* 2020, 76, 105109.

[S14] S. Kim, Y. Lee, H.-D. Kim, S.-J. Choi, *NPG Asia Materials* 2020, 12, 1.

[S15] F. Molina-Lopez, T. Gao, U. Kraft, C. Zhu, T. Öhlund, R. Pfattner, V. Feig, Y. Kim, S. Wang, Y. Yun, *Nature communications* 2019, 10, 2676.

[S16] W. Wang, S. Gao, Y. Li, W. Yue, H. Kan, C. Zhang, Z. Lou, L. Wang, G. Shen, *Advanced Functional Materials* 2021, 31, 2101201.

[S17] J. Yu, G. Gao, J. Huang, X. Yang, J. Han, H. Zhang, Y. Chen, C. Zhao, Q. Sun, Z. L. Wang, *Nature Communications* 2021, 12, 1581.

[S18] Y. He, S. Nie, R. Liu, S. Jiang, Y. Shi, Q. Wan, *Advanced Materials* 2019, 31, 1900903.

[S19] Y. R. Lee, T. Q. Trung, B.-U. Hwang, N.-E. Lee, *Nature Communications* 2020, 11, 2753.
